# Spray-Deposited TiO_2_–CuO Heterostructured Thin Films for Rifampicin Degradation and Solar Cell Application

**DOI:** 10.1021/acsomega.5c05919

**Published:** 2025-11-24

**Authors:** Marwa Jlaili, Wafa Naffouti, Neila Jebbari, Moez Hajji, Muzammil Hussain, Enrique Rodriguez Castellon, Pawan Kumar, Alberto Vomiero, Elisa Moretti, Kassa Belay Ibrahim, Najoua Turki-Kamoun

**Affiliations:** † Laboratoire de Physique de la Matière Condensée, Faculté des Sciences de Tunis, 37964 Université Tunis El Manar, 2092 Tunis, Tunisia; ‡ Department of Molecular Sciences and Nanosystems, 19047 Ca’ Foscari University of Venice, Via Torino 155, 30172 Venezia Mestre, Italy; § Departamento de Química Inorgánica, Cristalografía y Mineralogía, Facultad de Ciencias, Universidad de Málaga, Campus de Teatinos, 29071 Málaga, Spain; ∥ Division of Materials Science, Department of Engineering Sciences and Mathematics, Luleå University of Technology, 97187 Luleå, Sweden

## Abstract

TiO_2_, CuO, and TiO_2_–CuO heterostructures are commonly synthesized using hydrothermal or furnace-based methods, which often lack precise control over the thickness of the film. Moreover, their photocatalytic applications have mostly been limited to the degradation of conventional dyes such as methylene blue, methyl orange, and rhodamine B. Their use in degrading pharmaceutical pollutants remains largely unexplored. In this study, we report the synthesis of TiO_2_–CuO thin films via the spray pyrolysis method for the photocatalytic degradation of rifampicin (RMP), a pharmaceutical contaminant. The effects of varying the concentrations of TiO_2_ and CuO oxides in the sprayed solution at ratios (100:00, 75:25, 50:50, 25:75, and 0:100) on the thin films were explored and characterized with XRD, XPS, PL, and UV–vis Spectroscopy. TiO_2_ and CuO exhibited band gaps of 3.4 and 1.44 eV, respectively, while the optimized TiO_2_–CuO composite (T50C50) showed a slightly increased band gap of 1.47 eV, indicating strong interfacial coupling between the two oxides. Photoluminescence (PL) spectra indicated all samples’ emissions in both the UV and visible regions. The optimal ratio of TiO_2_ to CuO was determined to be 50:50 (referred to as T50C50). The photocatalytic degradation of RMP, a well-known antibiotic, under sunlight illumination demonstrated a high degradation rate of nearly 99% after 3 h. The influence of real operational parameters, such as pH, presence of scavengers, and catalyst dosage, has been investigated. Furthermore, simulations of solar cells utilizing TiO_2_–CuO absorber layers yielded a promising efficiency of approximately 22.5%. These findings indicate that TiO_2_–CuO heterostructured thin films have significant potential for optoelectronic applications and photocatalytic processes.

## Graphical abstract

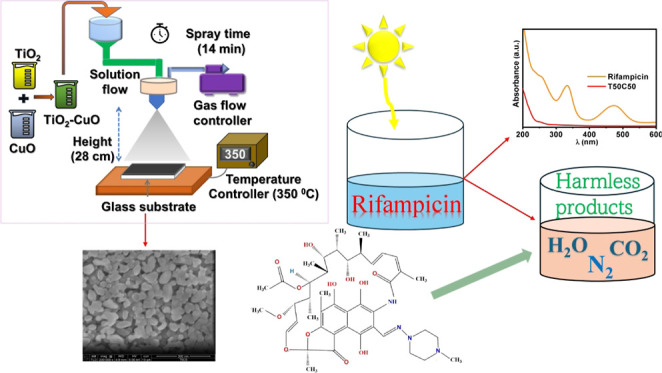

## 1 Introduction

Due to the drastic increase in human population and industrialization, we have witnessed a remarkable evolution in various aspects, like the energy crisis and environmental pollution.^1^ Among the variety of pollutants, organic compounds and residues from antibiotics stand out as significant contributors to water contamination.^2–4^ Antibiotics, hailed as marvels of modern medicine, have played a pivotal role in combating bacterial infections and saving countless lives.^5^ Yet, their extensive use has led to unintended consequences, including the presence of antibiotic residues in water bodies. Among these antibiotics, RMP, renowned for its efficacy against tuberculosis and other bacterial infections, is particularly noteworthy.^6–8^ The contamination of water sources with antibiotic residues poses a multifaceted challenge, encompassing ecological, public health, and environmental concerns. Consequently, innovative approaches are imperative to mitigate this issue effectively.^9^ Enter heterogeneous photocatalysis, a promising technology in the realm of water purification.^10^ This process involves the utilization of photocatalysts to degrade organic pollutants under light irradiation, offering a sustainable and efficient means of water treatment.^11^

Several studies have reported the application of advanced oxidation processes (AOPs) for RMP degradation using various material designs; however, the potential of oxide–oxide structures remain largely unexplored. Duarte et al. have demonstrated that electrochemical Fenton oxidation with different electrodes can achieve removal efficiencies of 43–46%.^12^ Liu et al. reported 79.9% degradation using rGO@nFe/Pd catalysis, which increased to 85.7% when combined with the Fenton reaction.^13^ Khataee et al. demonstrated that ultrasonic-assisted ZrO_2_-tuff oxidation reached 83% removal efficiency.^15^ Furthermore, metal oxide materials, including TiO_2_, ZnO, CuO, and WO_3_, have been widely explored as photocatalysts for the degradation of antibiotics in water treatment applications.^16–18^ However, their practical use is often limited by several intrinsic drawbacks. A major limitation is their restricted light absorption; for example, TiO_2_ and ZnO possess wide band gaps (∼3.2 eV), which confine their photocatalytic activity primarily to the ultraviolet (UV) region of the solar spectrum, thus limiting efficiency under natural sunlight. Although WO_3_ and CuO can absorb visible light to some extent due to their narrower band gaps, they still suffer from rapid recombination of photogenerated electron–hole pairs, leading to low quantum efficiency. Additionally, some of these oxides, such as ZnO and CuO, are prone to photocorrosion, which compromises their long-term structural stability and reusability. Furthermore, these materials generally exhibit low selectivity and poor degradation efficiency in complex real wastewater environments, where competing species can interfere with light absorption or deactivate active sites. These limitations collectively hinder the widespread application of metal oxide-based photocatalysts in the efficient and sustainable removal of antibiotic contaminants.^19^

To address these limitations, various strategies, including elemental doping to modify band gaps, surface modification to improve charge separation, and the incorporation of carbon-based materials such as graphene or carbon dots to facilitate electron transport, are explored.^19^ Among these approaches, the formation of heterostructures between two metals through the synergistic coupling of different metal oxides has emerged as one of the most promising solutions. This strategy enables efficient separation and transfer of photogenerated charge carriers by creating internal electric fields or built-in potential gradients at the interface of the coupled semiconductors. The resulting heterojunctions facilitate directional charge migration and suppress electron–hole recombination, thereby significantly enhancing photocatalytic activity under a broader range of light wavelengths. Moreover, heterostructure formation allows the complementary properties of individual oxides, such as the visible light response of CuO or WO_3_ and the stability of TiO_2,_ to be effectively combined, offering a more robust and versatile photocatalyst system for the degradation of antibiotics in complex aqueous environments.

In addition to photocatalytic degradation, we are also exploring the application of solar cells. As humanity evolves, the urgency for sustainable energy sources grows, particularly given the finite nature of traditional fossil fuels. Clean energy alternatives, such as solar cells, have gained significant attention as potential solutions.^20–22^ However, existing solar cell technologies face numerous challenges that require ongoing improvement.^23^ Among these, copper indium gallium selenide (CIGS) solar cells stand out as a promising option. Despite their relatively high efficiency and flexibility, CIGS solar cells have notable drawbacks, including the scarcity and toxicity of indium, along with a complex manufacturing process that limits scalability and raises production costs.^24^

The use of CuO as an absorber layer in solar cells has been widely investigated; however, the reported devices still suffer from low power conversion efficiencies. For example, Kidowaki et al.^25^ have achieved an efficiency of only 1.6%, while Jeong et al.^26^ reported 2.69%. Minami et al.^27^ obtained a maximum of 6.94%, and Hsueh et al.^28^ reported just 2.34%. Despite these efforts, the performance of CuO-based solar cells remains far below that of other established photovoltaic technologies.^29^ Therefore, there is an urgent need to develop new absorber layers that can overcome these challenges and advance solar cell technology toward a cleaner and more sustainable future. Based on this, herein, we design a TiO_2_–CuO heterostructure to harness their synergistic interaction for enhanced photocatalytic degradation of antibiotics.

## 2 Experimental Section

### 2.1 TiO_2_–CuO Thin Film Synthesis

TiO_2_–CuO heterostructured thin films were deposited on glass substrates by the spray pyrolysis technique, as shown in Figure 1a. All required precursors have been purchased from Sigma-Aldrich with high purity (>99%), ensuring the fabrication of good-quality thin films. Before the deposition, the glass substrates were ultrasonically cleaned in double-distilled water for 15 min. Then, substrates were rinsed using ethanol to remove any impurities. Finally, we kept all cleaned slides in the oven at 60 °C for drying. Then, TiO_2_ and CuO solutions were prepared independently. First, a TiO_2_ material solution was prepared by adding 2.4 mL of titanium tetra isopropoxide (TTIP: C_12_H_28_O_4_Ti) in 54 mL of ethanol (C_2_H_6_O) solvent and by using acetylacetone (AcAc: C_5_H_8_O_2_, 3.6 mL) as the stabilizing agent. On the other hand, 100 mL of CuO solution was prepared by dissolving 0.2 M copper chloride (CuCl_2_·2H_2_O) in 51 mL of deionized water. During the synthesis, we also optimized the TiO_2_: CuO ratios by varying them as follows: 100:0, 75:25, 50:50, 25:75, and 0:100. The resulting solutions were sprayed through a nozzle positioned at 28 cm above the glass substrate at a flow rate of about 7 mL min^–1^ and a substrate temperature of 350 °C, followed by annealing the obtained films in air at 500 °C for 2 h.

**Figure 1:**
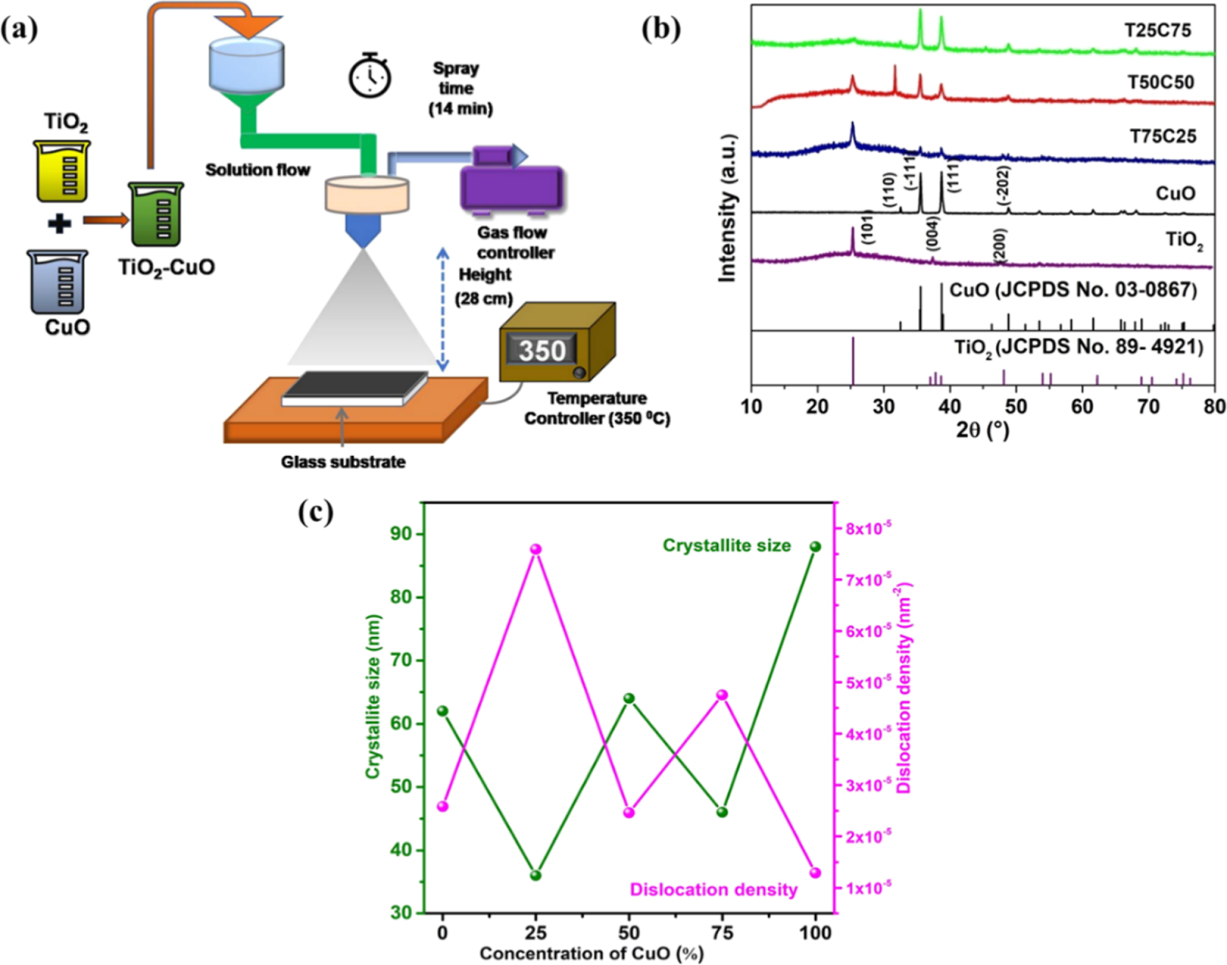
(a) Schematic illustration for the spray pyrolysis synthesis method, (b) X-ray diffraction patterns, (c) crystallite size and dislocation density of TiO_2_–CuO heterostructured thin films grown with different CuO contents.

### 2.2 Material Characterization Techniques

The Crystallographic structure for the TiO_2_–CuO thin film heterostructure was characterized by X-ray diffraction analysis (XRD), using the XPERT-PRO diffractometer system from 10 to 80°. The film thickness was estimated by the profilometry method (Bruker Dektak-XT profilometer). The surface morphology was investigated using a Sigma-VP Field Emission (FE-SEM) scanning electron microscope from Zeiss. Electronic property of the heterostructured material was studied by using X-ray Photoelectron Spectroscopy (XPS) was performed in a Physical Electronics spectrometer (PHI 5700), with X-ray source monochromated Mg Kα, 300 W, 15 kV, and 1253.6 eV. Optical characteristics were determined by UV-NIR spectrum with a PerkinElmer Lambda 950 spectrophotometer in the wavelength range of 250–2000 nm. Finally, photoluminescence (PL) spectra were recorded at room temperature using a PerkinElmer LS55 Fluorescence spectrometer with an excitation wavelength of about 300 nm.

### 2.3 Photocatalytic Degradation

In this study, RMP was used as a model antibiotic pollutant to evaluate photocatalytic degradation. The experiments began by immersing the samples in 50 mL of RMP solution for 60 min in the dark to establish adsorption–desorption equilibrium on the catalyst surface. Following this step, the TiO_2_–CuO thin films were exposed to sunlight for 120 min to assess their photocatalytic activity. During the irradiation period, aliquots were periodically collected to monitor the degradation process. Typically, Samples were taken at 30, 60, and 120 min, and their absorbance was measured using a spectrophotometer. These absorbance measurements enabled quantitative tracking of RMP degradation, where a decrease in absorbance indicated effective degradation, while stable or increasing absorbance values suggested incomplete degradation or persistence of the antibiotic.

### 2.4 Silvaco Atlas Ticad

In this work, the solar cell simulations were conducted using Silvaco Atlas Ticad. The simulation process began with defining the material parameters for each layer within the device structure. The electrical and optical properties for FTO, ZnO, and CdS were sourced from previous literature, while the parameters for the TiO_2_–CuO absorber layer were obtained from this work. In the device architecture, fluorine-doped tin oxide (FTO) and Mo serve as the front and back contact layers, enabling efficient charge collection. ZnO functions as the window layer, allowing light to pass through while providing electrical conductivity. CdS acts as the buffer layer, forming a junction with the absorber to facilitate charge separation while minimizing recombination losses at the interface. The TiO_2_–CuO layer serves as the primary absorber, capturing photons and generating electron–hole pairs.

The simulator generates the mesh, material, and structure files necessary for the main solver to model the photovoltaic behavior of the cell. Simulations were performed under standard AM 1.5 G illumination conditions with an incident power density of 0.1 W/cm^2^. Carrier recombination within the device was calculated using the Shockley–Read–Hall (SRH) recombination model to accurately reflect recombination mechanisms within the cell.

## 3 Results and Discussion

### 3.1 Structural Characterization of Thin Films

The crystalline and the structural information on heterostructure (different ratios of TiO_2_/CuO, 100:00, 75:25, 50:50, 25:75, and 0:100) were analyzed using X-ray diffraction (XRD) techniques. Figure 1b depicts a well-defined crystallinity for pure TiO_2_, CuO nanoparticles, and TiO_2_–CuO heterostructure was found for all the diffraction patterns. Pure TiO_2_ nanoparticles exhibited distinctive peaks at 2θ = 25.20, 37.68, and 48.11° belong to the anatase phase, corresponding to the crystal planes of (101), (004), and (200) (JCPDS No 89-4921). Monoclinic CuO has a tetragonal crystal phase, and the crystal planes of the main diffraction peaks at 32.38, 35.7, and 38.79° belong to (110), (−111), and (111), respectively. The diffraction angles were precisely matched with JCPDS 17-0923. The typical XRD diffractograms of TiO_2_–CuO heterostructure thin films showed well-defined peaks of TiO_2_ along with CuO at 2θ = 35.76° and 38.79^°,^ which can be ascribed to the (−111) and (111) lattice planes of CuO. Similarly, the peaks at 2θ = 25.20, 37.68, and 48.11° belong to the anatase phase of TiO_2_, corresponding to the crystal planes of (101), (004), and (200). The shift in the (−111) plan and absence of any impurity indicate the successful fabrication of TiO_2_–CuO heterostructure materials.^30,31^

The average grain size (*D*) of the synthesized thin films was calculated using the Debye–Scherrer formula, which utilizes the X-ray wavelength (λ = 1.540 Å), full width at half-maximum (fwhm), Bragg’s diffraction angle (θ), and a constant (*K* = 0.9). As can be seen in Figure 1c, the crystalline size of the synthesized samples was determined and found to vary depending on the composition. Pure TiO_2_ exhibited a crystalline size of 62 nm, while the TiO_2_–CuO composites showed different sizes based on their TiO_2_-to-CuO ratios. The T75C25 sample displayed a smaller crystalline size of 36 nm, whereas T50C50 and T25C75 exhibited sizes of 64 and 46 nm, respectively. Among the composites, T50C50 showed the largest crystalline size, indicating enhanced crystal growth at this composition. In comparison, pure CuO showed the largest overall crystalline size of 88 nm. These results indicate that the incorporation of CuO into TiO_2_ significantly influences the crystallite growth and structural properties of the composites. This suggests that T50C50 has relatively lower crystal defects and better structural quality.^32^

We believe that the grain size of the thin film significantly influences photocatalytic performance. While smaller grains offer larger surface areas, they also introduce more grain boundaries that act as trapping sites for charge carriers, potentially reducing photocatalytic efficiency. Conversely, larger grains like those in the T50C50 sample reduce grain boundaries, enhance diffusion pathways, and offer improved crystallinity. Structural parameters summarized in Table 1, such as dislocation density (δ) and microstrain (ε), derived from grain size and fwhm, were lowest for the T50C50 sample (2.46 × 10^–5^ nm^–2^ and 5.43 × 10^–4^, respectively), further affirming its superior structural integrity compared to other TiO_2_–CuO heterostructured thin films.

**Table 1:** Samples’ Nomination, Crystallite Size (*D*) Variation, Dislocation Density (δ_dis_), Microstrain (ε), and Film Thickness Variation of TiO_2_–CuO Heterostructured Films

sprayed volume (mL)	*V*_TiO2_	*V*_CuO_	sample	*D* (nm)	δ_dis_ (10^–5^ nm^–2^)	ε (10–4)	thickness (nm)
100	100	0	TiO_2_	62	2.58	5.56	509
	75	25	T75C25	36	7.59	9.54	1038
	50	50	T50C50	64	2.46	5.43	872
	25	75	T25C75	46	4.75	7.55	992
	0	100	CuO	88	1.29	3.93	657

### 3.2 Morphology and Elemental Analysis

SEM images, represented in Figure 2, are used to examine the morphology of the T50C50 thin film. In Figure 2a,b, done for two scales, 1 μm and 300 nm, it is evident that the T50C50 heterostructured thin film has a smooth surface, indicating the excellent quality of our thin layers and their suitability for optoelectronic applications. This smooth surface is a positive indication of film uniformity and lack of defects. A smooth surface can, indeed, be beneficial for photocatalysis in materials science due to several reasons. First, it reduces electron–hole recombination by providing fewer defect sites, thus allowing for more efficient charge carrier migration. Second, smooth surfaces enhance light harvesting by minimizing light scattering and reflection, leading to increased absorption of incident light and higher overall efficiency in photocatalytic reactions. Additionally, smooth surfaces promote better contact between the photocatalyst and reactants, facilitating faster reaction rates and more effective pollutant degradation.^33^

**Figure 2:**
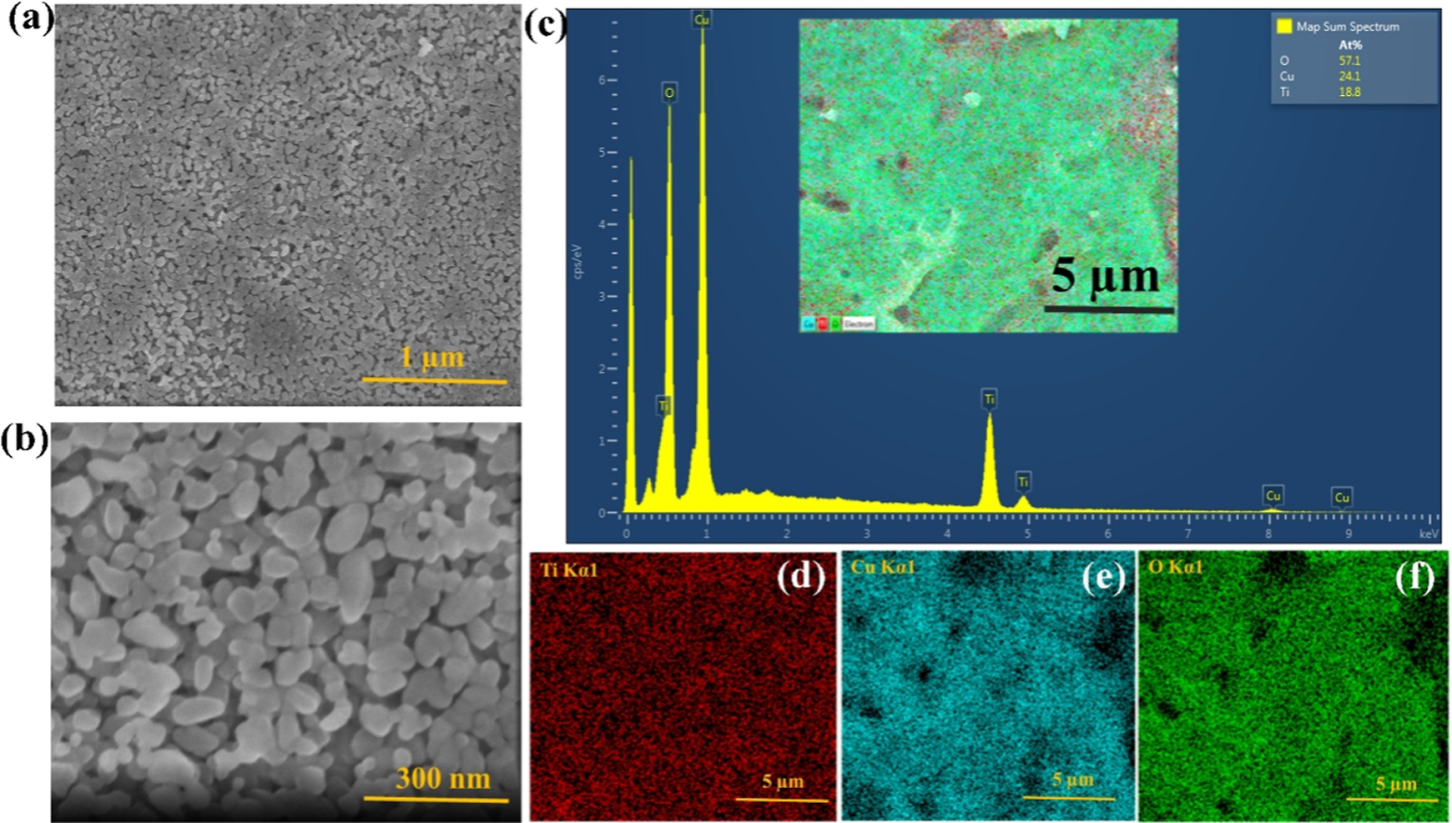
(a,b) SEM image at different scale bars. (c) Elemental mapping of (d) Ti, (e) Cu, and (f) O of T50C50 thin films.

EDS spectra show the elemental composition of the T50C50 thin film. The spectra, displayed in Figure 2c–f, confirms the presence of Ti, Cu, and O, elements in the TiO_2_–CuO composite uniformly distributed across the sample surface. Furthermore, our data illustrates that these elements are evenly dispersed throughout the thin film, indicating homogeneity in their spatial distribution. This uniformity is crucial for ensuring consistent material properties and performance in various applications, such as electronic devices, sensors, and catalysis.^34^

### 3.3 Sample Thickness Measurements

We all know that sample thickness plays a crucial role in photocatalytic degradation by influencing light absorption, charging carrier transport, and surface reaction efficiency. An optimally thick photocatalyst ensures sufficient light absorption to generate electron–hole pairs while minimizing recombination losses, as overly thick samples can hinder charge transport due to longer diffusion paths, leading to increased recombination.^35^ Conversely, overly thin layers may not absorb enough light to drive the reaction effectively. Additionally, excessive thickness can limit the diffusion of reactants and products to and from the active surface sites, reducing the overall degradation efficiency. Therefore, optimizing the photocatalyst thickness is essential to balance light absorption, charge mobility, and surface accessibility for maximum photocatalytic performance.^36^ Therefore, herein we calculate the film thickness (*t*) of TiO_2_–CuO heterostructured samples using the SEM cross-section. As depicted in Figure 3a, the T50C50 heterostructure material shows an average thickness of about 0.896 μm. Furthermore, this parameter is also measured and confirmed using the profilometry technique Figure 3b. The pure CuO thin film shows a thickness of 657 nm, which increases with an increase in concentration of CuO and TiO_2_ (T50C50) to 872 nm. All the results obtained are summarized in Table 1.

**Figure 3:**
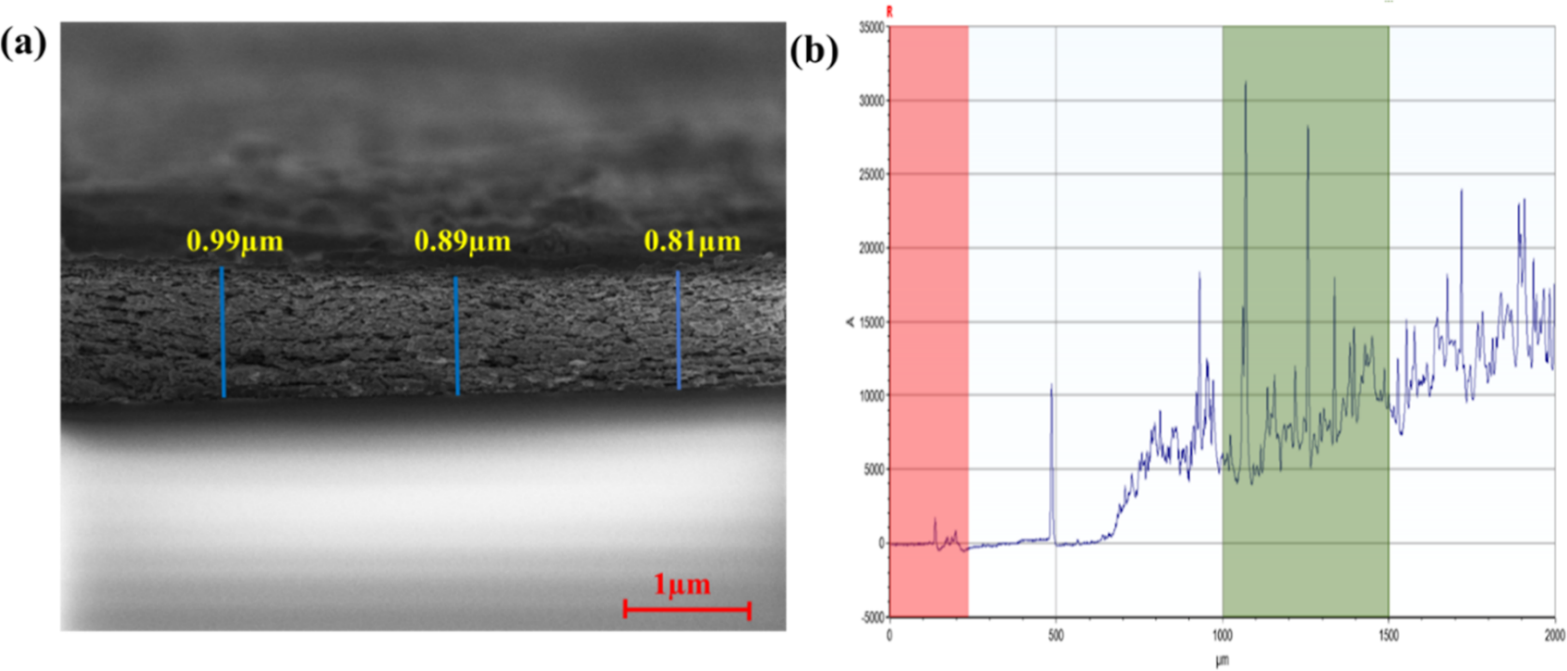
(a) T50C50 mixed oxide heterostructured thin film SEM cross-section and (b) profilometry analysis.

XPS surface analysis was used to more accurately investigate the effect of TiO_2_ on the electronic environment of CuO samples.^37^ For this purpose, the T50C50 as an optimized sample is used for XPS analysis. Figure 4a represents the wide range XPS spectra of T50C50. As expected, Cu, Ti, and O elements on the surface of the TiO_2_–CuO composites in their respective binding energy. The core level Cu 2p spectra XPS (Figure 4b) showed the presence of Cu 2p_3/2_ electronic states at 932.6 eV, representing the presence of CuO in the chemical state Cu^2+^. The other two peaks in the region of ∼940–945 eV correspond to the Cu 2p_3/2_ and shakeup satellites that appear when Cu^2+^ arises from energy losses due to electron excitations during photoemission. The XPS spectra of Ti 2p exhibited peaks at 457 and 464.0 eV, depicted in Figure 4c, which are attributed to the Ti 2p_3/2_ and Ti 2p_1/2_ spin orbital splitting, confirming the presence of Ti in a Ti^4+^ chemical state. The deconvoluted XPS spectrum of O 1s (Figure 4d) reveals three distinct peaks at 529.8, 531.2, and 532.8 eV, corresponding to lattice oxygen in TiO_2_ (77%), oxygen vacancies or carboxylate species (13%), and hydroxyl groups (C–OH, 10%), respectively. The predominant peak at 529.8 eV confirms the presence of well-crystallized TiO_2_ with a high proportion of lattice oxygen, while the shoulder at 531.2 eV indicates surface defects such as oxygen vacancies and chemically adsorbed carboxylate groups. The peak at 532.8 eV is attributed to hydroxyl functionalities, likely originating from adsorbed moisture or surface-bound hydroxyl groups.

**Figure 4:**
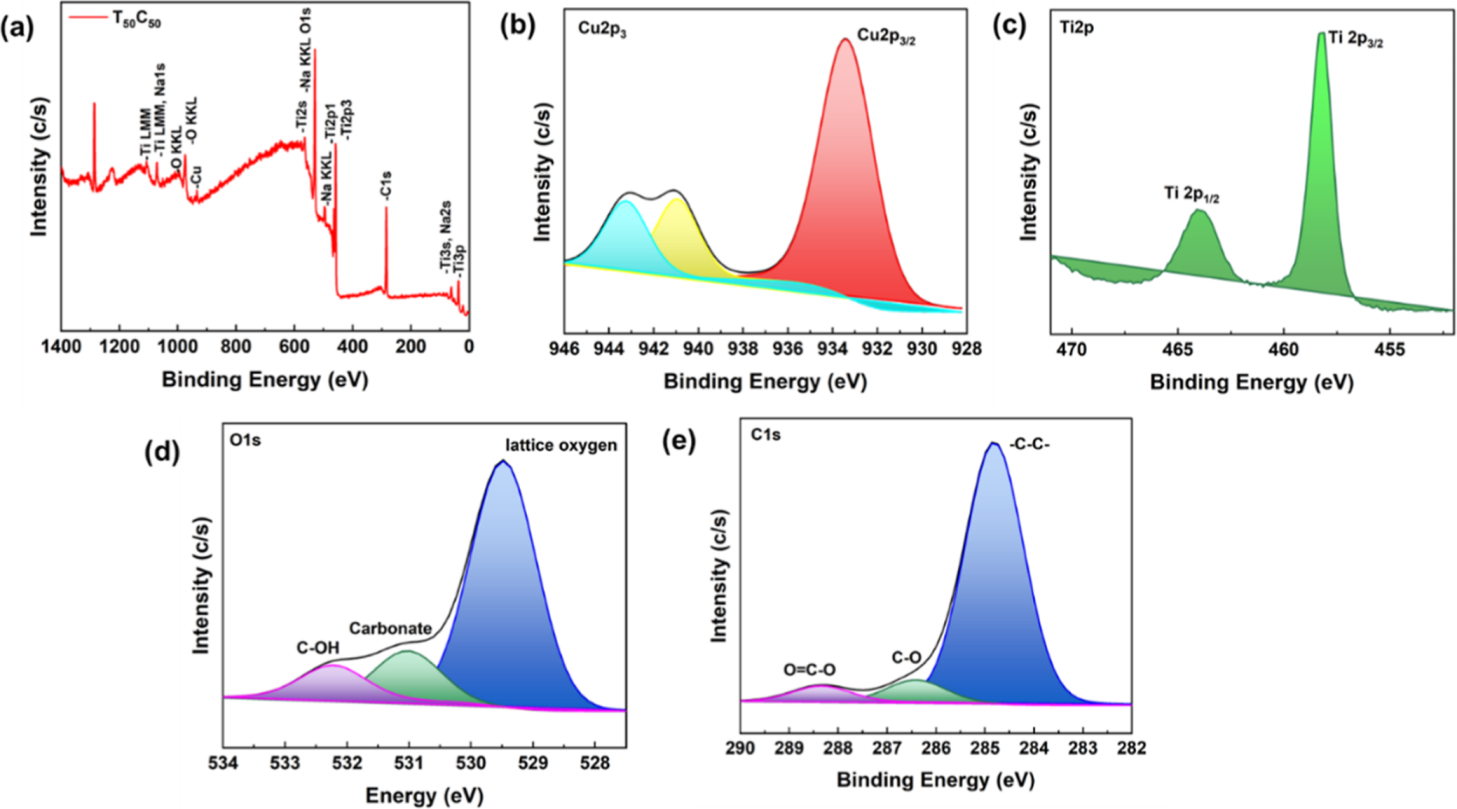
XPS spectra of T50C50 thin films (a) wide range spectra, (b) Cu 2p, (c) Ti 2p, (d) O 1s, and (e) C 1s spectra.

Similarly, the C 1s spectrum (Figure 4e) exhibits three main components: a dominant peak at 284.8 eV (87%), which is attributed to adventitious carbon species and sp^2^/sp^3^ hybridized carbon bonds (−C–C–, −C=C−); a secondary peak at 286.2 eV (8%), corresponding to C–OH groups; and a higher binding energy peak at 288.5 eV (5%), which is assigned to carboxylate species. These results confirm the presence of surface organic residues and functional groups, potentially introduced during synthesis or sample handling. The existence of M–O–C (M is Ti or Cu) related species suggests a successful interfacial interaction, supporting the formation of heterostructure thin film.^38^

### 3.4 Optical Studies

To investigate the optical properties of TiO_2_–CuO mixed oxide heterostructured thin films, UV–visible–NIR spectrophotometry was employed. The transmission spectra T­(λ) of films with varying TiO_2_ and CuO content are displayed in Figure 5a, covering the spectral range of 250–2000 nm. For pure TiO_2_ thin films, high transmittance exceeding 80% is observed in the visible region. A sharp absorption edge appears near 360 nm, corresponding to the optical band gap of TiO_2_, which is estimated to be approximately 3.40 eV, consistent with previous reports.^39^ Additionally, the presence of interference fringes in both the visible and near-infrared regions indicates good surface quality and uniform film thickness. In contrast, CuO thin films exhibit very low transmittance in the visible region, with the absorption edge located around 860 nm. This corresponds to an optical band gap of approximately 1.44 eV, aligning well with earlier studies.^40^ The high intrinsic absorption of CuO in the visible range makes it a promising material for photodetectors and absorber layers in optoelectronic applications.

**Figure 5:**
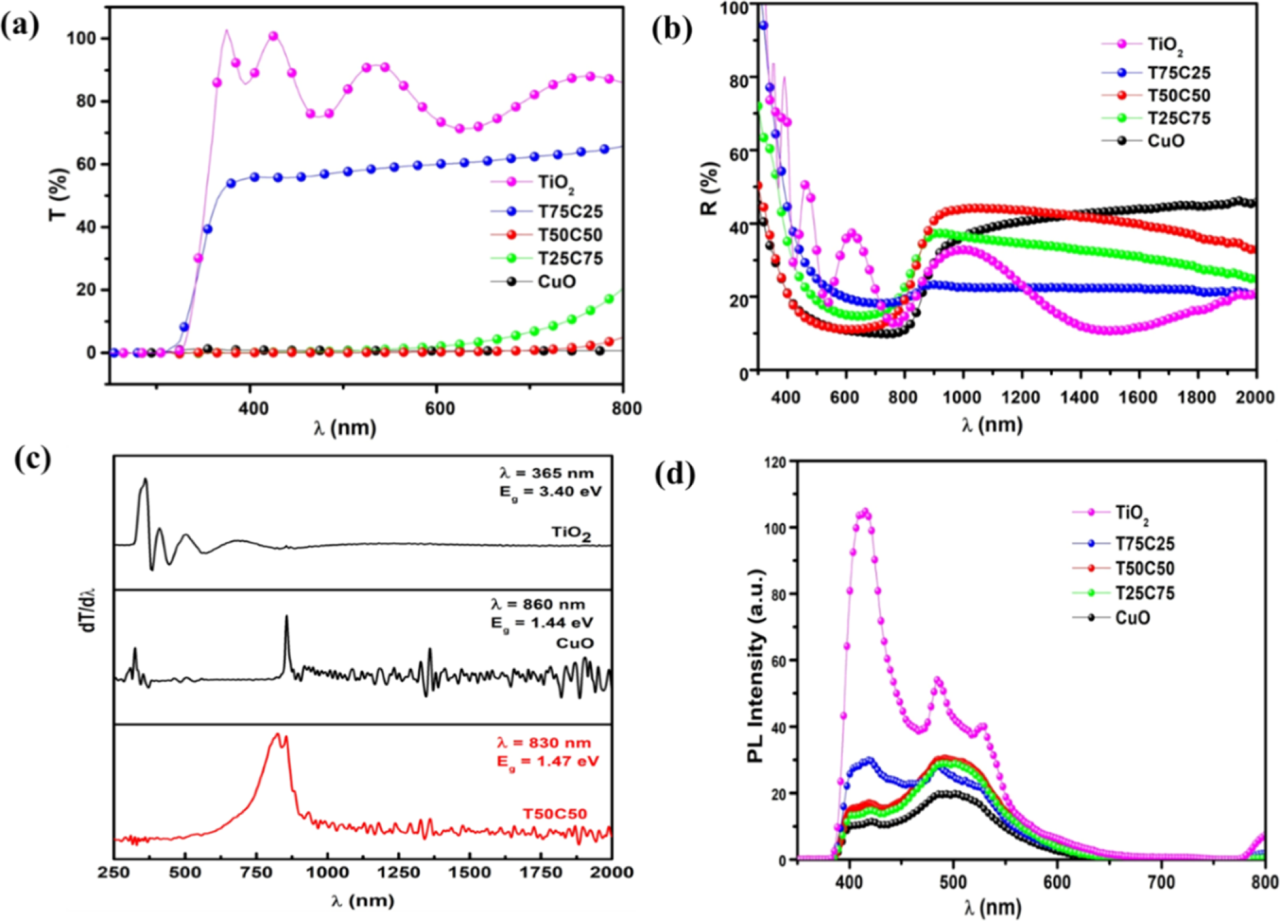
(a) Optical transmittance (*T*) (b) reflectance spectrum (*R*) of TiO_2_–CuO heterostructured thin films grown at various concentrations of TiO_2_ and CuO oxides (c) variation of d*T*/dλ as a function of λ for TiO_2_, T50C50 and CuO samples and (d) photoluminescence (PL) spectra of TiO_2_–CuO heterostructured thin layers grown for different contents of TiO_2_ and CuO.

As shown in Figure 5a, introducing CuO into the TiO_2_ matrix significantly reduces the transmittance of the mixed oxide films in the visible region. Increasing the CuO content leads to a pronounced redshift of the absorption edge, indicating a substantial reduction in optical band gap energy.^41^ The transmission spectra of TiO_2_-dominant films consistently show high transmittance (>80%) in the visible region and a sharp absorption edge toward shorter wavelengths, characteristic of the anatase phase. Interference fringes further confirm the uniformity, smoothness, and low scattering loss of these films, suggesting their suitability as optical windows in photovoltaic devices. In contrast, CuO thin films display near-zero transmission in the visible range with an absorption edge near 800 nm, again affirming their strong light-absorbing properties, making them excellent candidates for use as absorber layers in solar cells.^42^ With increasing CuO content in the TiO_2_–CuO heterostructures, a further decrease in transmission and a shift of the absorption edge to higher wavelengths are observed. Figure 5b shows the reflection spectra of the films deposited at different TiO_2_/CuO ratios via spray pyrolysis. All samples exhibit similar trends: a decrease in reflection in the UV region, followed by a relatively constant reflectance (∼20%) across the visible range.

For detailed optical characterization, the band gap energy (*E*_g_) was estimated using the Tauc plot method from the differential transmission spectra (d*T*/dλ).^43,44^ As can be seen from Figure 5c, the most intense peaks in these curves correspond to the band gap of the materials. As can be seen from Table 2, the optical band gap energies of the synthesized samples vary notably with composition. Pure TiO_2_ exhibited a wide band gap of 3.40 eV, while pure CuO showed a much narrower band gap of 1.44 eV. For the TiO_2_–CuO composites, a progressive decrease in band gap was observed with increasing CuO content, indicating strong interfacial interaction and coupling between the two oxides. Specifically, T75C25 showed a band gap of 3.36 eV, whereas T50C50 and T25C75 exhibited significantly lower values of 1.47 and 1.50 eV, respectively. Among the composites, T50C50 presented the most pronounced band gap reduction, suggesting that this composition provides the optimal balance for enhanced charge transfer and visible-light absorption efficiency. This result is also in agreement with previous reports.^45^

**Table 2:** Optical Band Gap Energy (*E*_g_) of TiO_2_–CuO Heterostructured Thin Films

sample	*E*_g_ (eV)
TiO_2_	3.40
T75C25	3.36
T50C50	1.47
T25C75	1.5
CuO	1.44

Photoluminescence (PL) spectroscopy is a crucial analytical technique in materials science, providing valuable information about the optical properties and electronic structure of various materials. Figure 5d depicts PL spectra of both pure CuO and TiO_2_ compounds as well as their composite (TiO_2_–CuO) mixed oxide, highlighting distinctive features essential for understanding their behavior. The PL spectrum of pristine TiO_2_ reveals three major peaks at approximately 425 nm (2.91 eV), 486 nm (2.55 eV), and 530 nm (2.33 eV).^45^ The peak located at around 425 nm is commonly linked to excitonic emissions, indicating the recombination of bound electron–hole pairs, and is influenced by factors such as crystal structure, doping, or defects in the TiO_2_. The 486 nm peak, located at the blue-green region, may also be associated with excitonic transitions, possibly arising from specific defect states or surface imperfections in the TiO_2_ lattice. Meanwhile, the 530 nm peak, found in the green region, is typically attributed to defect-related emissions, such as those originating from oxygen vacancies or titanium interstitials. For CuO, the PL spectrum exhibits three emission peaks centered at around 404.60 (3.06 eV), 418.71 (2.96 eV), and 496.51 nm (2.49 eV).^46^ The 404.60 nm peak likely originates from deep defect states within the CuO lattice, including vacancies or impurities, resulting in ultraviolet photon emission.^31^ 418.71 nm peak is thought to arise from shallower defects or surface states caused by crystal imperfections, emitting photons in the visible range.^47^ Lastly, the 496.51 nm peak is associated with lower-energy transitions related to defect-induced or surface states, further shaping the overall PL behavior of CuO.^48^ Together, these spectral features provide critical insights into the optical characteristics and potential functional applications of TiO_2_, CuO, and their composite materials in areas like optoelectronics and photocatalysis. In the case of TiO_2_–CuO heterostructured thin films, photoluminescence (PL) spectra exhibit a diverse array of emissions within the visible range, underscoring the complex optical behavior of these composite materials. Across all deposited films, characteristic emissions in violet, blue, and red regions emerge, with peaks situated around 405, 486, 497, and 796 nm, respectively. Notably, the emissions at 405 and 497 nm are attributed to CuO emission peaks, indicative of the presence and activity of CuO within the heterostructured structure. Conversely, the peak observed at 486 nm aligns with TiO_2_ emission peaks, providing compelling evidence for the successful synthesis of TiO_2_–CuO mixed oxide. This distinct spectral fingerprint not only underscores the coexistence of TiO_2_ and CuO components but also highlights the potential synergistic effects and novel functionalities that arise from their integration, offering promising avenues for advanced optoelectronic and catalytic applications.

### 3.5 Photocatalytic Activity

RMP represents a significant pollutant in water bodies, posing environmental and health risks due to its persistence and potential toxicity. RMP, an antibiotic employed in medical treatments, can contaminate water sources through industrial discharge and improper disposal. Addressing the challenge of their removal demands efficient catalytic approaches. Titanium dioxide-copper oxide (TiO_2_–CuO) emerges as a promising catalyst for the degradation of both pollutants.^49^ To evaluate the photocatalytic activity of the as-prepared TiO_2_–CuO heterostructured thin films, which were elaborated with different copper concentrations, the photodegradation of RMP, a well-known antibiotic and a typical pollutant in the textile and pharmaceutical industries, was investigated in water under sunlight illumination. Figure 6b shows the UV–vis absorbance spectrum of RMP aqueous solution with and without TiO_2_–CuO heterostructured thin layers after 3 h under sunlight illumination. It is evident that the characteristic absorption peak located at 475 nm decreases rapidly with the extension of exposure time, illustrating the removal of the dyes by the photocatalyst, and then the degradation efficiency increases (Figure 6c).

**Figure 6:**
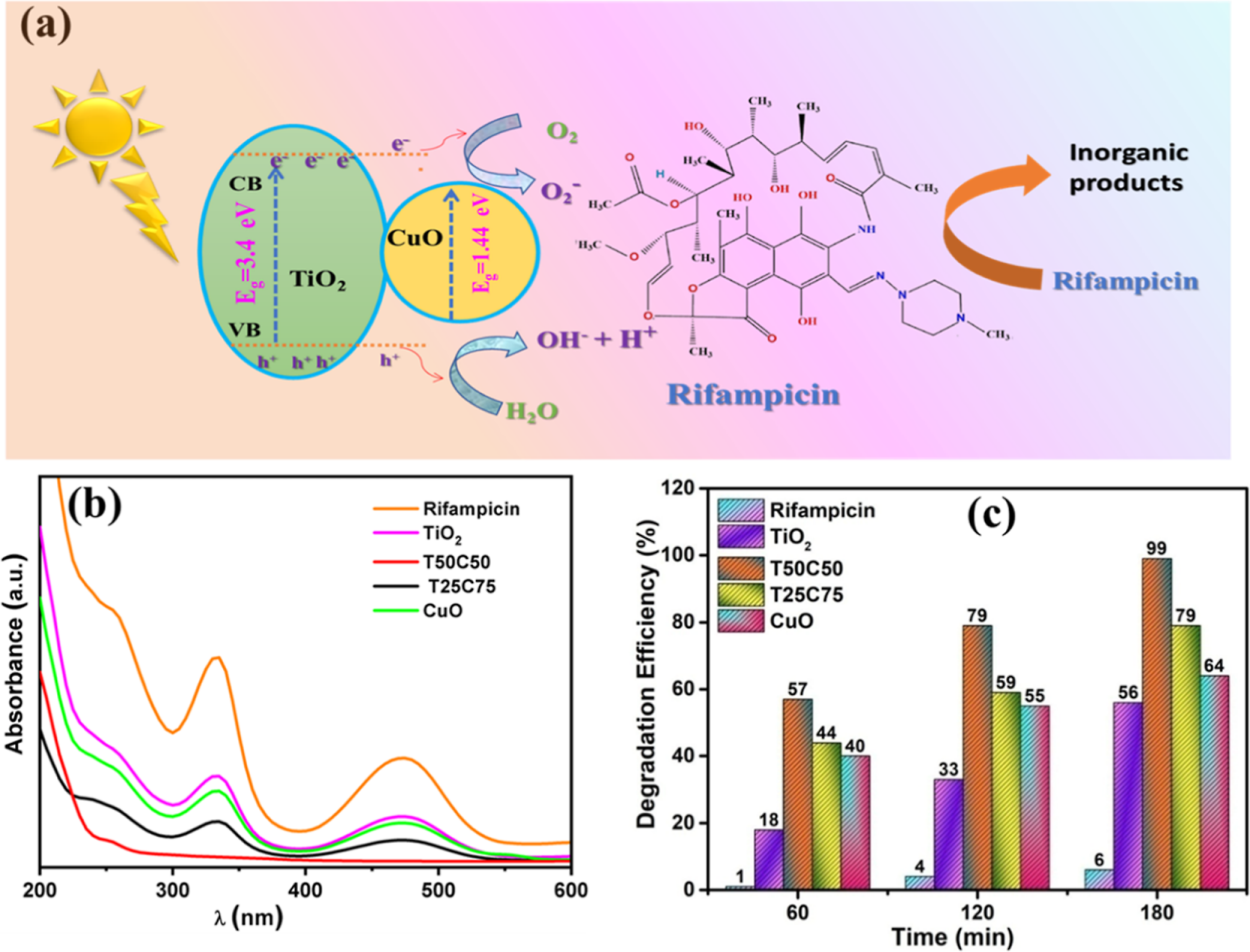
(a) Schematic diagram of photocatalytic degradation mechanism using TiO_2_–CuO. (b) Absorbance spectra, (c) photodegradation efficiency of RMP for different photocatalysts.

The rate constant is calculated using the following relation^50^

$$C/C_{0}=\exp\left(-kt\right)$$

where *k* denotes the rate constant, *C* and *C*_0_ depict the initial and final concentration of dye, respectively. The rate constants of the RMP degradation without and with the growth layers are about 0.007, 0.12, 0.003, 0.27, and 0.16 for TiO_2_, CuO, T75C25, T50C50, and T25C75 thin layers, respectively. Photodegradation efficiency of RMP with and without a photocatalyst thin layer is calculated using the following relation^51^

$$efficiency\;\left(\%\right)=\frac{C_{0}-C}{C_{0}}\times 100$$

Under sunlight illumination, TiO_2_–CuO heterostructured thin films, especially the T50C50 composition, demonstrate superior photocatalytic activity compared to pure TiO_2_ or CuO films. While TiO_2_ is limited to UV absorption and CuO suffers from high electron–hole recombination, their combination enables broader light absorption, improved charge separation, and more active sites for pollutant degradation. The T50C50 sample also benefits from an optimized grain size with fewer crystal defects and a higher surface area, further enhancing its performance. These synergistic effects make TiO_2_–CuO heterostructures a promising and efficient strategy for solar-driven environmental remediation, surpassing the performance of individual oxides reported in earlier studies. The presented work has been compared with other similar studies done before (Table 3).

**Table 3:** Comparison Table for Photodegradation of RMP by Different Nanocomposites

nanocomposites	pollutants	light	time (min)	% of degradation	ref.
Ti/Ru_0.3_Ti_0.7_O_2_	RMP	UV	200	43	^12^
rGO@nFe/Pd	RMP	visible	150	79	^13^
Cu_2_O–Ag–CaWO_4_ (CAC)	RMP	visible	100	96	^14^
US/ZrO	RMP	visible	100	85	^15^
TiO_2_–CuO	RMP	sun	180	99	this work

#### 3.5.1 Effect of Catalyst Dosage

Figure 7a presents the photocatalytic degradation of Rifampicin using TiO_2_–CuO thin films at different catalyst dosages: 5 mg, 10 mg, 20 mg, 30 mg, 50 mg, and 100 mg. The graph shows the variation of ln­(*C*/*C*_0_) over time, confirming that the degradation follows a pseudo-first-order kinetic model. As shown in the figure, increasing the catalyst dosage generally enhances the degradation rate, as indicated by the progressively steeper negative slopes of the curves.^52^ The best performance is observed at a catalyst dosage of 20 mg, which exhibits the most pronounced decrease in ln­(*C*/*C*_0_) over time. This indicates that 20 mg of TiO_2_–CuO provides the most efficient degradation of Rifampicin under the tested conditions. This improvement can be attributed to the increased number of active sites available on the catalyst surface at this dosage, promoting better photon absorption and more effective generation of electron–hole pairs. These charge carriers play a crucial role in forming reactive oxygen species (ROS), such as hydroxyl radicals (^•^OH), responsible for the breakdown of Rifampicin molecules.

**Figure 7:**
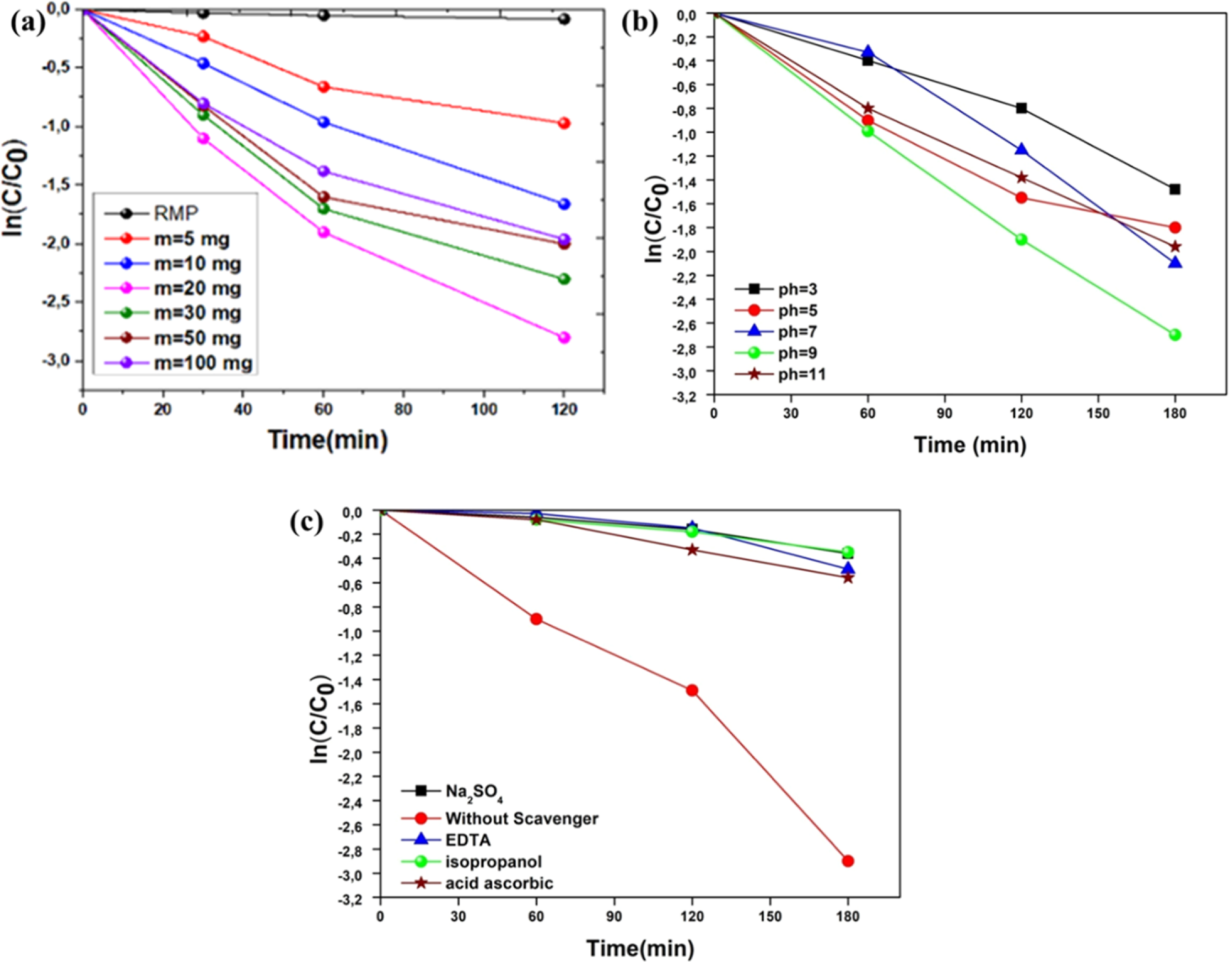
Effect of different adsorption parameters (a) catalyst dosage, (b) pH, (c) scavengers.

However, when the dosage is further increased to 30 mg and 50 mg, a slight decrease in photocatalytic efficiency is observed compared to 20 mg, though the performance remains relatively high. This suggests that while these dosages still provide effective degradation, the system begins to experience minor light scattering or shielding effects, limiting the full activation of the photocatalyst. At 100 mg, a more pronounced decline in efficiency occurs, likely due to excessive catalyst loading, which leads to particle aggregation or sedimentation and reduces the effective surface area exposed to light. Furthermore, excessive catalyst concentration can cause significant light scattering and shielding, limiting light penetration into the suspension. Therefore, 20 mg remains the optimal catalyst dosage, offering the best balance between active surface area and light utilization, while 30 mg and 50 mg still maintain near-optimal performance before a clear decline is seen at higher loadings.^53^

#### 3.5.2 Effect of pH

Figure 7b illustrates the influence of solution pH on the photocatalytic degradation of RMP using TiO_2_–CuO thin films. The degradation behavior, represented by the change in ln­(*C*/*C*_0_) over time, was studied at pH values of 3, 5, 7, 9, and 11. The data clearly show that the degradation rate is strongly dependent on pH, with the most efficient degradation occurring at pH 9. At acidic conditions (pH 3 and pH 5), the degradation efficiency is relatively low. This can be attributed to the positive surface charge of the photocatalyst under acidic pH, which may lead to electrostatic repulsion with the cationic form of RMP, limiting its adsorption on the catalyst surface. Additionally, the formation of hydroxyl radicals, which are crucial for photocatalytic oxidation, is less favorable in highly acidic media.^54^

In contrast, as the pH increases to neutral and alkaline conditions, especially at pH 9, the degradation efficiency significantly improves. At this pH, the surface of the TiO_2_–CuO photocatalyst becomes negatively charged, which enhances the adsorption of RMP and promotes the generation of reactive oxygen species, particularly hydroxyl radicals (^•^OH), due to increased availability of hydroxide ions (OH^–^). These radicals play a key role in breaking down the antibiotic molecules. The maximum degradation rate at pH 9 suggests that this is the optimal condition for efficient photocatalysis under the studied parameters.^55^ At very high pH (pH 11), a slight decline in performance is observed, which could be due to the instability of the photocatalyst or reduced photoactivity in extreme alkaline conditions. This highlights the importance of maintaining a moderately alkaline environment to achieve optimal degradation efficiency.

#### 3.5.3 Effect of Scavengers

Figure 7c shows the effect of various scavengers on the degradation of RMP using TiO_2_–CuO thin films. The addition of specific scavengers helps to identify the dominant reactive species involved in the degradation process. Compared to the control sample without scavenger (red line), the addition of Na_2_SO_4_ (electron scavenger), EDTA (hole scavenger), isopropanol (^•^OH radical scavenger), and ascorbic acid (superoxide radical scavenger) significantly reduced the degradation efficiency. The strong inhibition observed with isopropanol and ascorbic acid indicates that hydroxyl radicals (^•^OH) and superoxide radicals (O_2_^•–^) play a major role in the photocatalytic process. The reduction in activity with EDTA also highlights the involvement of photogenerated holes (h^+^). These results suggest that multiple reactive species contribute to RMP degradation, with hydroxyl and superoxide radicals being the most influential.

### 3.6 Degradation Mechanism of RMP

The mechanism of coupled oxide semiconductors, such as TiO_2_–CuO, for pollutant degradation involves multiple redox stages. As illustrated in Figure 8, the exposure of the Rifampicin (RMP) solution containing TiO_2_–CuO thin films (specifically the T50C50 composition) to solar irradiation leads to the excitation of electrons from the valence band of TiO_2_ to its conduction band, leaving behind positively charged holes (h^+^). These photoinduced charge carriers (h^+^/e^–^ pairs) initiate a series of oxidative and reductive reactions at the catalyst surface.^56^ The electrons in the conduction band react with dissolved oxygen molecules to produce superoxide radicals (^•^O_2_^–^), while the photogenerated holes oxidize surface hydroxyl groups or adsorbed water to generate highly reactive hydroxyl radicals (^•^OH). These reactive oxygen species (ROS) are primarily responsible for the breakdown of RMP into various intermediates and eventually into mineralized end products.

**Figure 8:**
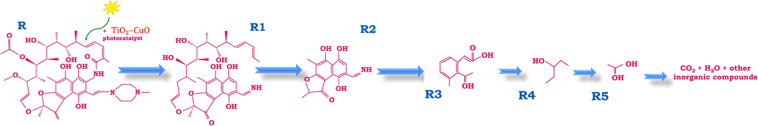
Photocatalytic degradation pathway of RMP.^57^

According to the LC–MS analysis and as presented in Figure 8, the degradation of RMP (*m*/*z* = 882) proceeds through a sequence of oxidative steps. The parent molecule first undergoes N–N bond cleavage and dehydroxylation, forming the intermediate R1 (*m*/*z* = 781). Further oxidation and demethylation reactions yield R2 (*m*/*z* = 270), corresponding to partial fragmentation of the rifampicin chromophore. Subsequent ring-opening and decarboxylation steps lead to smaller organic intermediates such as R3 (*m*/*z* = 195), R4 (*m*/*z* = 88), and R5 (*m*/*z* = 61). These compounds, consisting mainly of short-chain organic acids and alcohols, are eventually mineralized to CO_2_, H_2_O, and other inorganic ions, confirming the progressive degradation pathway. The T50C50 sample exhibits the highest photocatalytic activity, achieving approximately 99% degradation efficiency under solar irradiation. This superior performance can be attributed to efficient charge separation and interfacial electron transfer between TiO_2_ and CuO, which minimizes recombination and enhances ROS production. It is important to note that this proposed mechanism provides a plausible interpretation of the degradation pathway but may vary depending on different experimental conditions such as pH, catalyst composition, and irradiation intensity.^57,58^

### 3.7 Solar Cell Simulation

Before conducting experimental measurements, the SnO_2_: F/TiO_2_/ZnO/CdS/T50C50/Mo solar cell structure was simulated using Silvaco Atlas by solving the semiconductor continuity and Poisson equations as defined in

$$\Delta V=-\frac{q}{\varepsilon }\left[p-n+N^{d}-N^{a}+N_{t}\right]$$

where *V* is the electrostatic potential, *q* is an electron charge, ε is the permittivity of the material, *N*_a_ is the acceptor doping density, *N*_d_ is the donor doping density, *p* is the hole density, *n* is the electron density and *N*_t_ is the acceptor-type and donor-type defect density.

The following equations define the continuity equations

$$-\frac{1}{q}\frac{dJ_{n}}{dx}=G\left[X\right]-R_{n}$$

$$-\frac{1}{q}\frac{dJ_{p}}{dx}=G\left[X\right]-R_{p}$$

where *J*_*p*_ is the hole current density, *J*_*n*_ is the electron current density.

Next, based on the Schottky equation, the [*J*–*V*] system equation can be written as follows

$$J=I_{ph}-I_{0}\exp\left[\frac{qV}{\alpha KT}-1\right]$$

$$I_{sc}=I_{ph}$$

$$V_{oc}=\frac{\alpha KT}{q}\ln\left[\frac{I_{ph}}{I_{0}}\right]$$

$$I_{0}=q\left[\frac{D_{n}n_{i,p^{2}}}{L_{n}N_{a}}+\frac{D_{p}n_{i,p^{2}}}{L_{p}N_{d}}\right]$$

where *n*_i,p_ and *n*_i,n_ are the intrinsic carrier densities of holes and electrons in the layers, *D*_p_ and *D*_n_ are the hole and electron diffusion coefficients, *L*_p_ and *L*_n_ are hole and electron diffusion lengths, respectively, *I*_SC_ is the short circuit, *V*_oc_ is the open voltage circuit, *I*_0_ the dark current, *k* is the Boltzmann constant and *T* is the temperature.

Finally, we define the fill factor (FF) and the efficiency (ρ) by the following equations^59^

$$FF=\frac{P_{\max}}{V_{oc}I_{sc}}$$

$$\rho =\frac{P_{\max}}{P_{in}}$$

Considering its high absorbance and suitable band gap, this composition seeks our attention to consider as a promising alternative to conventional absorber materials like CIGS and CdTe in photovoltaic applications.^60^ As depicted in Figure 9a,b, we provide a visual representation of the structure and mechanism of our simulated solar cell. Our solar cell structure consists of front and back contacts made of fluorine-doped tin oxide (FTO) and molybdenum (Mo), respectively. The window layer is composed of ZnO, while the buffer layer is made of CdS.

**Figure 9:**
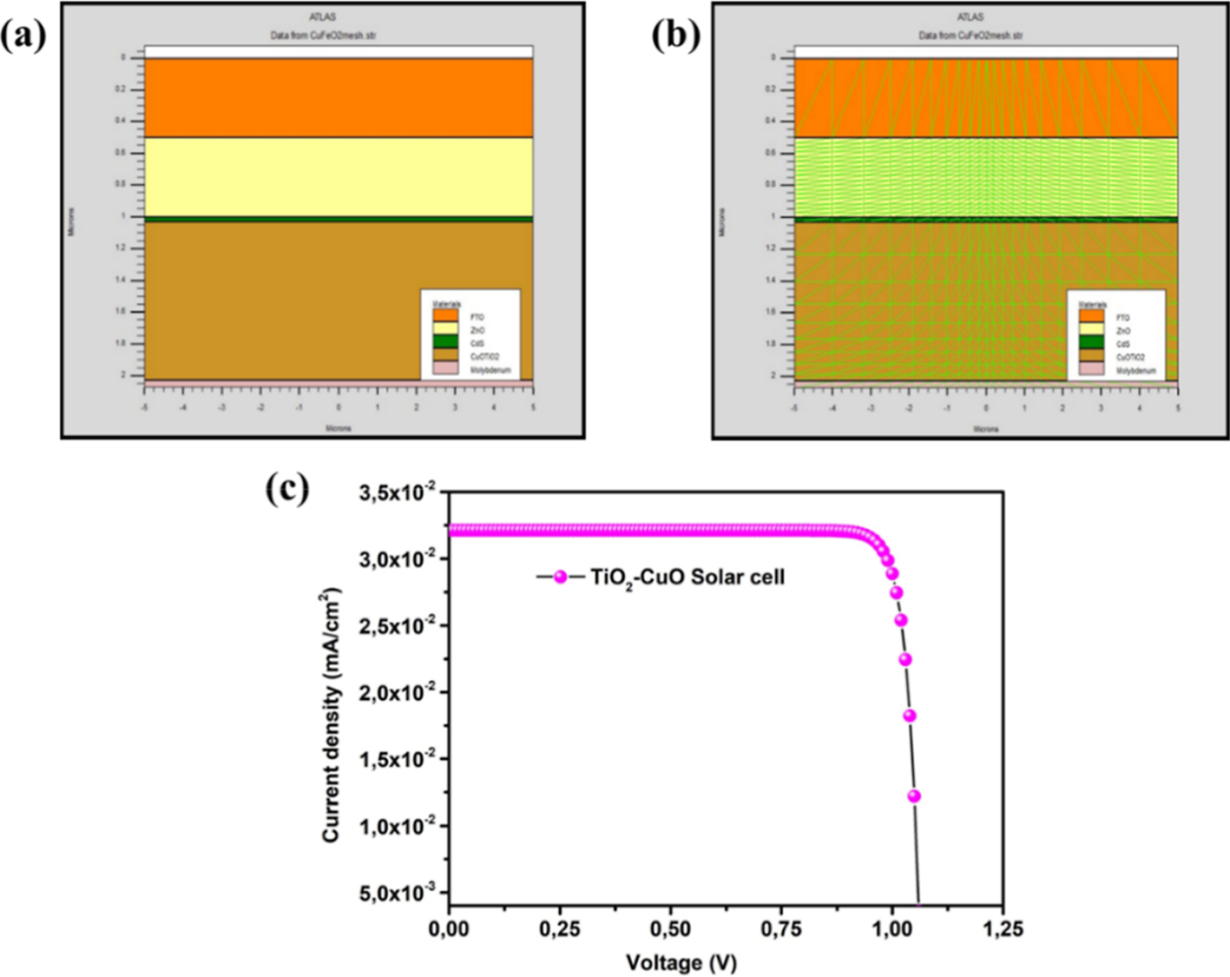
Solar cell simulation results: (a) solar cell structure, (b) mesh result, and (c) *J*–*V* curve.

The key component of our solar cell is the absorber layer, which is composed of our T50C50 thin films. These thin films have been carefully designed to have an optimal solar cell conversion gap energy of about 1.5 eV. The *J*–*V* curve, depicted in Figure 9c, provides valuable insights into the performance of our solar cell. The results indicate a short current density (*J*_SC_) of about 32.66 mA, an open voltage (*V*_OC_) of 1.15 V, and a fill factor (FF) in the order of 80.26. Furthermore, the efficiency ρ of our solar cell is calculated to be 22.5%. These impressive results validate the high efficiency of our thin film as an absorber layer and position it as a promising candidate for applications in solar cells, replacing copper Indium gallium selenide (CIGS) and cadmium telluride (CdTe) thin films. Overall, this study serves as a comprehensive illustration of the structure and performance of our simulated solar cell, highlighting the potential of our T50C50 thin films as a key component in achieving efficient solar energy conversion. These results can be explained by band gap engineering of our solar cell structure and the optimized value of our absorber layer to be equal to 1.47 eV.

## 4 Conclusion

In conclusion, using the Spray pyrolysis technique, we have successfully investigated the growth of TiO_2_–CuO heterostructured thin films with varying concentrations of TiO_2_ and CuO. The solution concentration of both TiO_2_ and CuO oxides was found to have a significant impact on the structural, morphological, and optical properties of sprayed heterostructured thin films. Furthermore, the application of all-grown TiO_2_–CuO heterostructured thin films in RMP degradation has been investigated. We have demonstrated that the highest efficiency of RMP, about 99% over 3 h, is obtained in the case of the T50C50 sample. Additionally, the deposited films were studied as an absorber layer SnO_2:_ ZnO/CdS/T50C50/Mo solar cell using the Silvaco package, showing a promising efficiency of 22.5%. These findings highlight the potential of TiO_2_–CuO heterostructured thin films for various applications, particularly in catalysis and as an absorber layer in solar cells.
